# Post-Weaning Protein Malnutrition Increases Blood Pressure and Induces Endothelial Dysfunctions in Rats

**DOI:** 10.1371/journal.pone.0034876

**Published:** 2012-04-18

**Authors:** Aucelia C. S. de Belchior, Jhuli K. Angeli, Thaís de O. Faria, Fabiana D. M. Siman, Edna A. Silveira, Eduardo F. Meira, Carlos P. da Costa, Dalton V. Vassallo, Alessandra S. Padilha

**Affiliations:** 1 Departamento de Fisiologia e Farmacologia, Universidade Federal de Pernambuco, Recife, Pernambuco, Brazil; 2 Programa de Pós-Graduação em Ciências Fisiológicas, Universidade Federal do Espírito Santo, Vitória, Espírito Santo, Brazil; 3 Escola de Ensino Superior da Santa Casa de Misericórdia de Vitória, EMESCAM, Vitória, Espírito Santo, Brazil; Université de Montréal, Canada

## Abstract

Malnutrition during critical periods in early life may increase the subsequent risk of hypertension and metabolic diseases in adulthood, but the underlying mechanisms are still unclear. We aimed to evaluate the effects of post-weaning protein malnutrition on blood pressure and vascular reactivity in aortic rings (conductance artery) and isolated-perfused tail arteries (resistance artery) from control (fed with Labina®) and post-weaning protein malnutrition rats (offspring that received a diet with low protein content for three months). Systolic and diastolic blood pressure and heart rate increased in the post-weaning protein malnutrition rats. In the aortic rings, reactivity to phenylephrine (10^−10^–3.10^−4^ M) was similar in both groups. Endothelium removal or L-NAME (10^−4^ M) incubation increased the response to phenylephrine, but the L-NAME effect was greater in the aortic rings from the post-weaning protein malnutrition rats. The protein expression of the endothelial nitric oxide isoform increased in the aortic rings from the post-weaning protein malnutrition rats. Incubation with apocynin (0.3 mM) reduced the response to phenylephrine in both groups, but this effect was higher in the post-weaning protein malnutrition rats, suggesting an increase of superoxide anion release. In the tail artery of the post-weaning protein malnutrition rats, the vascular reactivity to phenylephrine (0.001–300 µg) and the relaxation to acetylcholine (10^−10^–10^−3^ M) were increased. Post-weaning protein malnutrition increases blood pressure and induces vascular dysfunction. Although the vascular reactivity in the aortic rings did not change, an increase in superoxide anion and nitric oxide was observed in the post-weaning protein malnutrition rats. However, in the resistance arteries, the increased vascular reactivity may be a potential mechanism underlying the increased blood pressure observed in this model.

## Introduction

Proper nutrition in terms of quality and quantity is essential for the growth and development of organisms, including humans. Furthermore, nutritional deficiencies due to a decrease in or absence of the consumption of macro and micronutrients in food causes malnutrition in which the degree depends on the type of diet, age and the length of decrease consumption. Malnutrition is the result of an interaction of socioeconomic, political, cultural and environmental factors that more strongly affect children who live in extreme poverty [1].

Previous reports have shown that not only intrauterine malnutrition but also its occurrence during childhood might be a risk factor for the development of hypertension [2], [3]. Malnutrition is accompanied by increased blood pressure, and this increase could be due in part to an impairment of endothelial function and/or increased sympathetic activity [4], [5]. Some authors have shown that, during the period of malnutrition, the levels of circulating catecholamines increase [6], [7]. This could be one of the hypertensive mechanisms induced by malnutrition. However, other mechanisms, such as changes in endothelial function, could contribute to the development and/or maintenance of hypertension induced by malnutrition. Previous reports have shown that in rats intrauterine malnutrition increases the generation of superoxide anion by increasing NADPH oxidase activity, which is possibly induced by angiotensin II [5], [8]. However, these studies were conducted under intrauterine malnutrition. There are few studies that have investigated endothelial function in chronically malnourished rats. Moreover, there are also few studies evaluating models of malnutrition based on results of food consumption in populations.

In northeast Brazil, nutritional studies on the food consumption of the population allowed for the development of an experimental multideficient diet model, the regional basic diet (RBD) [9]. This diet is similar, in terms of quality, quantity and protein content to the diet that causes childhood malnutrition in Northeast Brazil [9]. Also, according to Teodosio et al. (1990), it is an unbalanced diet and low in some nutrients, mainly proteins. Both essential and non-essential amino acids are extremely limited in this diet, in addition to calories, fat, vitamins and minerals.

However, the factors that promote the development of hypertension associated with malnutrition are still unclear. Therefore, we evaluated the impact of the post-weaning malnutrition in rats on blood pressure, vascular reactivity of the rat tail vascular bed (resistance vessels) and aortic rings (conductance vessels).

## Materials and Methods

### Animals and experimental groups

The studies were performed on male Wistar rats. All experiments were conducted in compliance with the guidelines for biomedical research as stated by the Brazilian Societies of Experimental Biology and approved by the Ethics Committee on Animal Experimentation of the Biological Sciences Center of the Federal University of Pernambuco (Process Number 23076.008507/2007-16). All rats had free access to water and were fed rat chow *ad libitum*.

Animals were divided into two experimental groups: control (CT) and post-weaning protein malnutrition over 3 months. In the control group, the mothers were fed a chow diet during the pre-mating, mating, pregnancy and lactation. After weaning (21 days), the offspring had the same diet as their mothers (Labina®). In the post-weaning protein malnutrition group, mothers were fed a Labina® diet during the pre-mating, mating, pregnancy and lactation phases. The offspring received the RBD ration post weaning for 3 months.

### Diets

Two types of diets were used for this study. The diet used to induced malnutrition was the RBD (Table 1) as described previously [9]–[12]. The RBD consisting of beans (*Phaseolus vulgaris*), sweet *potatoes (Ipomea batatas)*, jerked beef and manioc flour *(Manihot esculenta)*. These components were macerated, molded into “pellets” and heated to 50°C. The RBD pellets providing a total of (g/g%): proteins 9, carbohydrates 78, lipids 1.1, fiber 7, minerals 4, sodium chloride 0.17 and Kcalorie 356. No vitamin supplement was added. Moreover, part of the diet was supplemented with 0.2% (g/g) sodium chloride. The control diet was a commercial diet (Labina®). The standard diet contains the following content (g/g%): protein 23, carbohydrates 41, lipids 2.5, fibers 9, minerals 8, sodium chloride 0.37 and Kcalorie 278 [10].

**Table 1:** Composition (g/g %) of the Regional Basic Diet (RBD) and the control diet.

Diets	Protein	Carbohydrates	Lipids	Vitamin Supplement	Minerals	Sodium	Fiber	Kcal/100
**RBD** 1	9	78	1.1	No	4	0.37	7	356
**Control** 2	23	41	2.5	Yes	8	0.37	9	278

1 According to the Laboratory of Experimentation and Analysis of Food (LEEAL), Nutrition Department, Federal University of Pernambuco.

2 As indicated by the manufacturer (Purina Agriband, Paulínia, SP, Brazil).

### Hemodynamic measurements

At the end of treatment of malnutrition (3 months), the rats were anesthetized with urethane (1.2 g/kg, i.p.), and a polyethylene catheter (PE50) filled with heparinized saline (50 U/mL) was introduced into the carotid artery to record arterial systolic (SBP) and diastolic blood pressure (DBP). Recordings were performed over 30 min with a pressure transducer (TSD 104A- Biopac) and with an interface and software for data collection (MP 30 Biopac Systems, Inc.; CA). The heart rate (HR) was determined from the intra-beat intervals.

### “In Vitro” Experiments

#### Isolated rat aorta preparation

After the hemodynamic measurements, rats were killed by exsanguination. The thoracic aortas were carefully dissected and separated from the connective tissue. For the reactivity experiments, the aortas were divided into cylindrical segments 4 mm in length. To analyze the isoforms of endothelial and inducible nitric oxide synthase protein expression levels, the arteries were rapidly frozen in liquid nitrogen and kept at −70°C until the day of analysis.

Segments of thoracic aorta were mounted in an isolated tissue chamber containing Krebs–Henseleit solution: 118 mM NaCl; 4.7 mM KCl; 23 mM NaHCO_3_; 2.5 mMCaCl_2_; 1.2 mM KH_2_PO_4_; 1.2 mM MgSO_4_; 11 mM glucose and 0.01 mM EDTA, gassed with 95% O_2_ and 5% CO_2_, and maintained at a resting tension of 1 g at 37°C as a previously described [13]. The isometric tension was recorded using an isometric force transducer (TSD125C, CA, U.S.A.) connected to an acquisition system (MP100 Biopac Systems, Inc., CA, U.S.A.).

After a 45 min equilibration period, the aortic rings were initially exposed twice to 75 mM KCl once to check their functional integrity and a second time to assess the maximal tension. Afterwards, the endothelial integrity was tested by adding acetylcholine (10 µM) to segments previously contracted with phenylephrine (1 µM). A relaxation equal to or greater than 90% was considered to be demonstrative of functional integrity of the endothelium. After a 45-min washout, concentration-response curves to phenylephrine were determined. Single curves were performed in each segment. The effects of the following drugs were evaluated: (1) a nonspecific NOS inhibitor N-nitro-L-arginine methyl ester (L-NAME, 100 µM), (2) a non-selective cyclooxygenase (COX) inhibitor (indomethacin, 10 µM), (3) an NADPH oxidase inhibitor (apocynin, 0.3 mM). These drugs were added to the bath 30 min before performing the phenylephrine curve.

The influence of the endothelium on the response to phenylephrine in the post-weaning protein malnutrition and control groups were investigated after performing a mechanical removal by rubbing the lumen with a needle. The absence of endothelium was confirmed by the inability of 10 µM acetylcholine to produce relaxation.

#### Western blot analyses

Proteins from homogenized arteries (80 µg) were separated by 10% SDS-PAGE. The proteins were transferred to nitrocellulose membranes that were incubated with mouse monoclonal antibodies for endothelial nitric oxide synthase (eNOS, 1∶250; Transduction Laboratories, Lexington, UK). After washing, the membranes were incubated with anti-mouse (1∶5000, StressGen, Victoria, Canada) immunoglobulin antibody conjugated to horseradish peroxidase. After thorough washing, the immunocomplexes were detected using an enhanced horseradish peroxidase/luminal chemiluminescence system (ECL Plus, Amersham International, UK) and film (Hyperfilm ECL International). Signals on the immunoblot were quantified with the National Institutes of Health Image V1.56 computer program. The same membrane was used to determine α-actin expression using a mouse monoclonal antibody (1∶5000, Sigma, USA).

#### Isolated rat tail artery preparation

Isolated rat tail arteries were used in this study as previously reported [14]. Briefly, after the hemodynamic measurements, the rats were heparinized (500 UI, *i.p*.). Ten minutes after the administration of heparin, 1 cm of the tail artery was dissected free and cannulated with an intracath (Nipro 24G ¾, Sorocaba, SP, BR) near the base of the tail. The vascular bed was flushed with Krebs-Henseleit buffer (KHB in mM) (NaCl: 120, KCl: 5.4, MgCl_2_: 1.2, CaCl_2_: 1.25, NaH_2_PO_4_: 2.0, NaHCO_3_: 27, glucose: 11, and EDTA: 0.03) and bubbled with 5% CO_2_ −95% O_2_ at 36±0.5°C. The tail artery was then severed from the body and placed in a tissue bath and perfused with KHB at a constant flow of 2.5 mL/min with a peristaltic pump (Milan, Colombo, PR, BR).

After a 45-min equilibration period, the experimental protocol was initiated. The mean perfusion pressure (MPP) was measured by using a pressure transducer (TSD104A, BIOPAC Systems, Inc., USA), and the data were recorded using an interface and software for data acquisition (model MP100A, BIOPAC Systems, Inc.). Because a constant flow was used, changes in the perfusion pressure represented changes in vascular resistance.

The following protocols were used to investigate the effects of post-leaning malnutrition on vascular reactivity to phenylephrine, acetylcholine and sodium nitroprusside. After a 45-min stabilization period, increasing doses of phenylephrine (0.001–300 µg as bolus injections of 100 µL) were administered into the perfusion medium in preparations from the controls and post-weaning protein malnutrition groups. The arteries were then maintained for a 30-min stabilization period. Then, the vasodilatation induced by acetylcholine (10^−10^–10^−3^ M) was evaluated in arteries previously contracted with potassium chloride (65 mM) added to the perfusion fluid. Finally, after a 30-min stabilization period, the dilatory responses to sodium nitroprusside (10^−10^–10^−3^ M) were determined in arteries previously contracted with potassium chloride (65 mM) added to the perfusion fluid.

### Statistical analysis

All values are expressed as mean ± S.E.M. The contractile responses to phenylephrine of the aortic rings are expressed as a percentage of the maximum response produced by 75 mM KCl added into the bath. Relaxation response to Acetylcholine (10 µM) was expressed as the percentage of relaxation of the maximal contractile response to phenylephrine (1 µM).

Results regarding the perfusion pressure measurements for the tail artery experiments are presented as the changes in the mean perfusion pressure after subtraction of the peak pressure from the baseline pressure. The concentration-responses curves to acetylcholine or sodium nitroprusside are expressed as the percentages of relaxation of the maximum contractile response to KCl 65 mM.

For each concentration-response curve or dose-response curve, the maximum effect (E_max_) and the concentration of agonist that produced 50% of the maximal response (log EC_50_) were calculated using non-linear regression analysis (GraphPad Prism Software, San Diego, CA). The sensitivity of the agonists is expressed as pD_2_ (−log EC_50_). To compare the effects of endothelium denudation or drug incubation on contractile responses to phenylephrine, some results were expressed as the differences of the area under the concentration–response curves (dAUC) in both experimental groups. These values indicate whether the magnitude of the effect of endothelial denudation or drug incubation were different in the control and post-weaning protein malnutrition groups.

For protein expression, the data are expressed as the ratio between signals on the immunoblot corresponding to the studied protein and α-actin. The results are expressed as the means ± SEM of the number of rats studied. Differences were analyzed using Student's *t*-test or one-way ANOVA followed by a Tukey test. *P<0.05* was considered to be significant.

### Drugs and reagents

L-phenylephrine hydrochloride, L-NAME, indomethacin, apocynin, acetylcholine chloride, urethane and sodium nitroprusside were purchased from Sigma-Aldrich (St. Louis, MO, USA). Salts and reagents used were of analytical grade from Sigma (St. Louis, MO, USA) and Merck (Darmstadt, Germany).

## Results

No difference in body weight between the groups was observed either at birth or 21 days after birth (Table 2). However, 90 days after birth, the body weight was lower in the post-weaning protein malnutrition rats compared to the controls (Table 2). These results demonstrated that the RBD was able to induce a loss in weight gain.

**Table 2:** Body weight (g) measurements of control (CT) and post-weaning protein malnutrition (Malnutrition) offspring.

	CT (N = 10)	MALNUTRITION (N = 10)
**Birth**	6.31±0.7	6.45±0.6
**Weaning (21^st^ day)**	59.6±2.9	58.2±1.4
**90^st^ day**	376±7.3	131±9.7*

Values are expressed as the mean ± SEM.

* *P*<0.05 Control *vs.* Malnutrition, Student's *t-*test.

In order to verify if the loss in weight gain induced by hypoproteic diet produces arterial pressures alterations, blood pressure and heart rate were measured. At the end of treatment of malnutrition (90 days), significant increases in systolic and diastolic arterial blood pressure and heart rate were observed in the post-weaning protein malnutrition group compared to the controls (Table 3). Therefore, these results demonstrated that the post-weaning protein malnutrition induces a weight defect and hypertension.

**Table 3:** Systolic (SBP, mmHg) and diastolic (DBP, mmHg) blood pressure and heart rate (HR, bpm) determination in the control (CT) and post-weaning protein malnutrition (Malnutrition) adult rats.

	CT (N = 10)	MALNUTRITION (N = 10)
**SBP (mmHg)**	102±4.8	118±4*
**DBP (mmHg)**	54±4	72±5.8*
**FC (bpm)**	302±9	328±11*

Values are expressed as the means ± SEM.

* *P*<0.05 Control *vs.* Malnutrition, Student *t*-test.

Blood pressure changes could occur by an increase of cardiac output or/and by increased vascular resistance. Therefore, to further investigate the underlying mechanisms that produced the increase in arterial pressure, the effect of post-weaning malnutrition on vascular reactivity was investigated in isolated aortic rings (conductance arteries) and in the tail vascular bed (resistance arteries). In aortic rings, malnutrition did not affect the maximal response to 75 mM KCl in segments with endothelium (CT: 2.74±0.12 g vs. post-weaning protein malnutrition: 2.38±0.06 g, n = 10) and after endothelium removal (CT: 2.62±0.12 g vs. post-weaning protein malnutrition: 2.50±0.20 g, n = 10). These results demonstrated that the maximal tension that developed in aortic rings of post-weaning protein malnutrition group compared to the controls were not different. After that, vasodilatation and vasoconstriction mechanisms were investigated in aortic rings from post-weaning malnutrition and controls rats.

**Figure 1:**
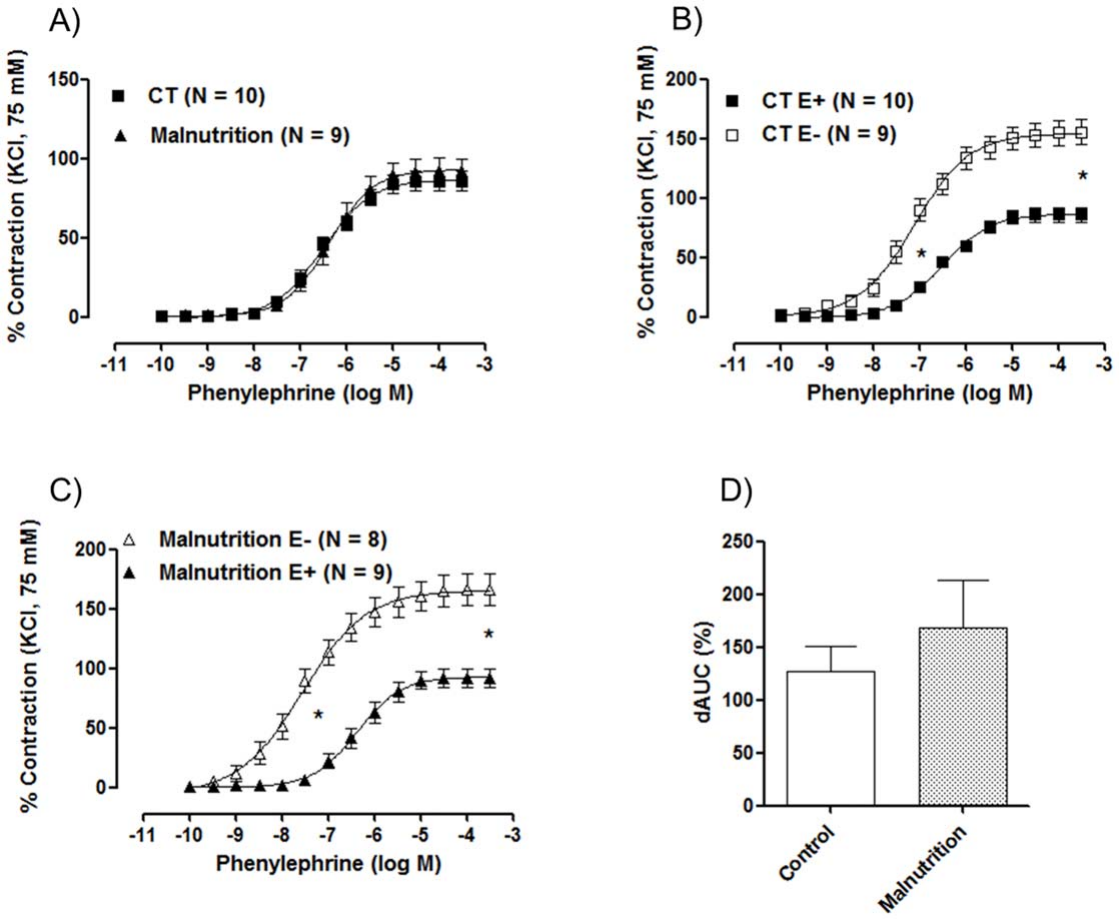
The effects of post-weaning protein malnutrition on the concentration-response curves of phenylephrine. (A) Concentration-response curve to phenylephrine in isolated aortic rings from control (CT) and post-weaning protein malnutrition (Malnutrition) groups. (B, C) Concentration-response curves to phenylephrine in isolated aortic rings from control (CT) and post-weaning protein malnutrition (Malnutrition) groups before (E+) and after removal of endothelium (E−). (C) Percent difference of the area under the curve (% dAUC) in vessels with endothelium intact (E+) and denuded (E−). The number of preparations is indicated in parenthesis. The results are expressed as the means ± SEM. * *P<0.05* for pD_2_ and R_max_: CT E+ *vs.* Ct E−; pD_2_: Malnutrition E+ *vs.* Malnutrition E− and dAUC% - CT *vs.* Malnutrition. Student's *t-*test.

The endothelium-dependent relaxation induced by acetylcholine in phenylephrine-contracted arteries was similar in aortic rings isolated from controls and post-weaning protein malnutrition (Rmax - CT: 93.86±2.7 g vs. post-weaning protein malnutrition: 100.05±1.12 g, n = 8), suggesting that the oxide nitric release induced by acetylcholine was not modified. The contractile response induced by phenylephrine did not change in aortic rings from post-weaning protein malnutrition rats compared to the control group (Figure 1A). The influence of the endothelium on the response to phenylephrine was investigated after its mechanical removal. Endothelium removal leads to hyper contraction and produced a left-shifted concentration-response curve to phenylephrine in aortic segments from both groups (Figure 1B and 1C), but this effect was not different, as shown by the differences of the area under the concentration–response curves (Figure 1D).

**Figure 2:**
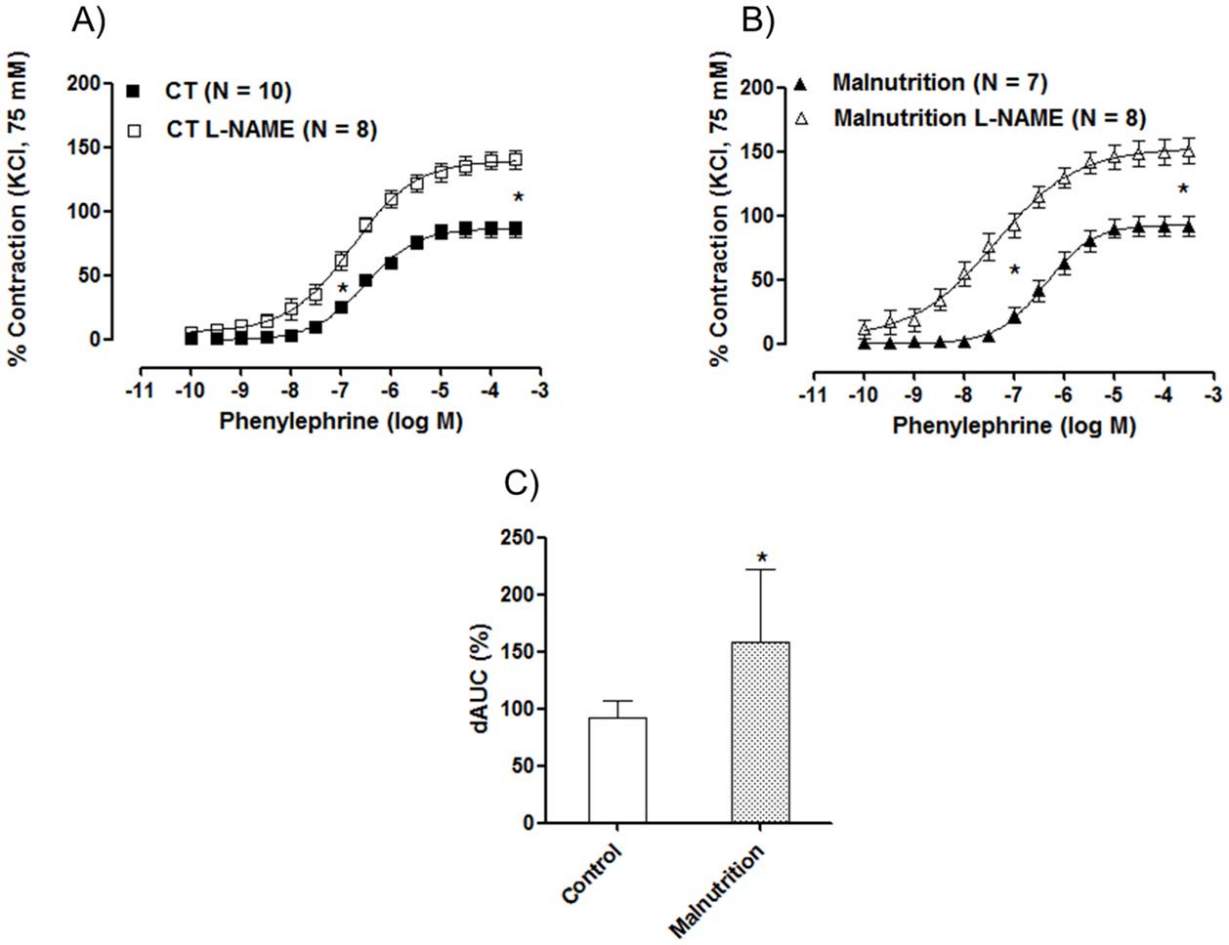
The effects of *N^G^*-nitro-L-arginine methyl ester (L-NAME, 100 µM) (A, B) on the concentration-response curve to phenylephrine in isolated aortic rings from control (CT) and post-weaning protein malnutrition (Malnutrition). (C) Comparison of the percent difference of the area under the curve (% dAUC) in vessels in presence or absence of L-NAME from control (CT) and post-weaning protein malnutrition (Malnutrition) groups. The number of preparations is indicated in parenthesis. The results are expressed as the means ± SEM. * *P*<0.05 for pD2 and R_max_: CT E+ vs. CT L-NAME; pD2 and R_max_: Malnutrition E+ vs. Malnutrition L-NAME and dAUC% CT *vs.* Malnutrition by Student's *t*-test.

**Figure 3:**
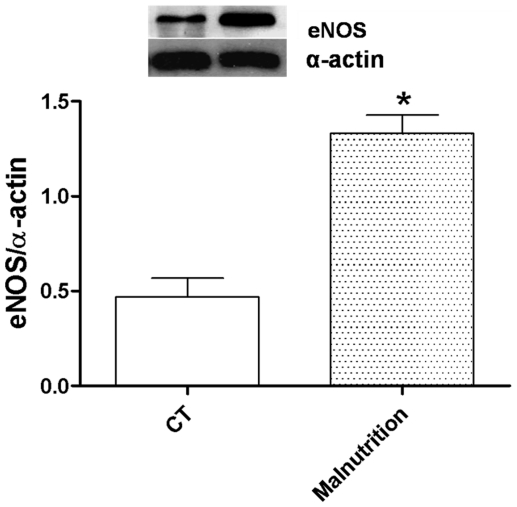
Densitometric analysis of the Western blots for endothelial NO synthase (eNOS) protein expression in the isolated aortic rings from Wistar rats (CT, N = 9) and post-weaning protein malnutrition (Malnutrition, N = 9). The number of preparations is indicated in parenthesis. Each bar represents means ± S.E.M as the ratio between the signal for the eNOS protein and the signal for α-actin. * *P<0.05* for CT *vs.* Malnutrition by Student's *t*-test. Representative blots are shown.

To investigate whether the nitric oxide release in response to phenylephrine in aortic rings from post-weaning protein malnutrition was altered, rings were incubated with L-NAME. L-NAME (100 µM) induces a hyper contraction and left-shifted the concentration-response curve to phenylephrine in the aortic segments from both groups (Figures 2A and 2B). However, these effects were greater in preparations from the post-weaning protein malnutrition rats compared to controls, as shown by the differences of the area under the concentration-response curves (Figure 2C). In accordance with these findings, eNOS was overexpressed in aortic rings from the post-weaning protein malnutrition rats (Figure 3). These results suggested that the post-weaning protein malnutrition increased the release of nitric oxide, probably by eNOS overexpression.

**Figure 4:**
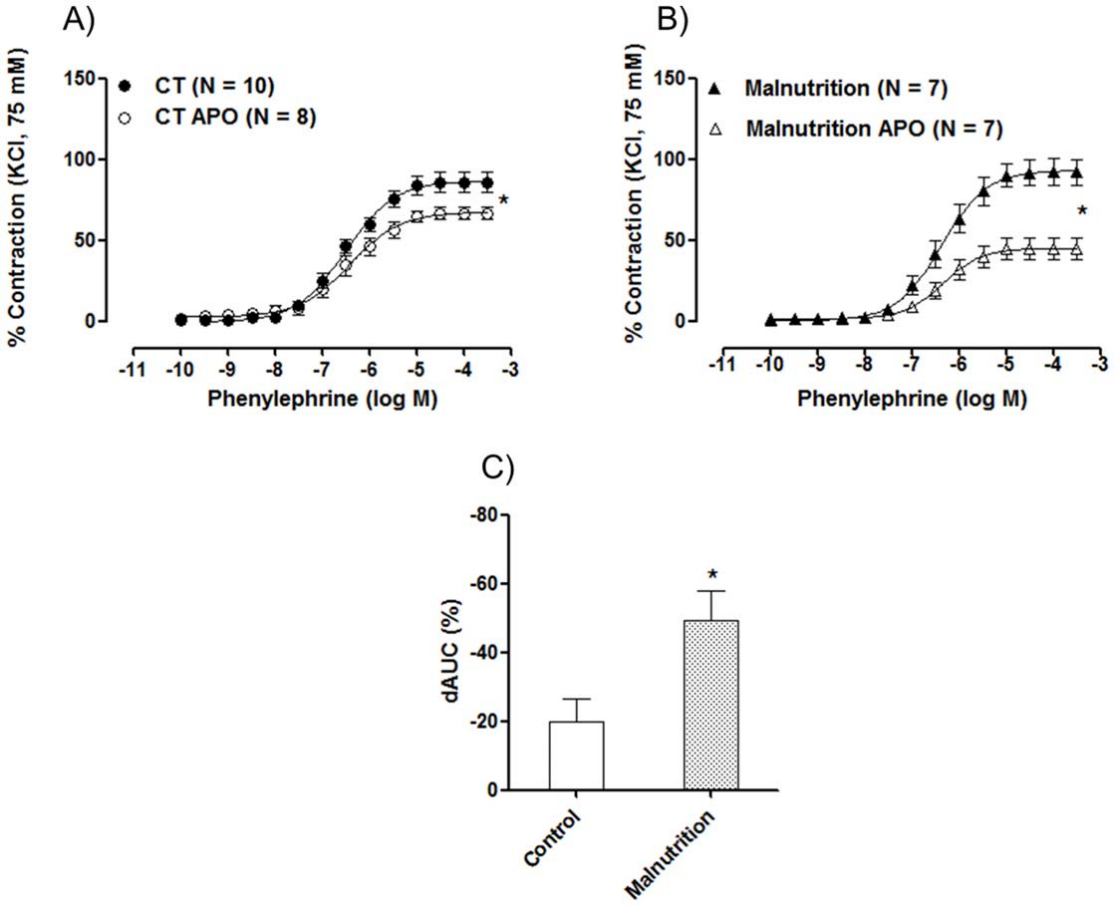
Concentration-response curve to phenylephrine in isolated aortic rings from (A) control (CT) and (B) post-weaning protein malnutrition (Malnutrition) rats before (E+) and after incubation with apocynin (APO). C) Comparison of the percent difference of the area under the curve (% dAUC) in vessels in presence or absence of apocynin from control (CT) and post-weaning protein malnutrition (Malnutrition) rats. The number of preparations is indicated in parenthesis. The results are expressed as the means ± SEM. * *P<0.05* for R_max_: CT E+ *vs.* CT APO; R_max_: Malnutrition E+ *vs.* Malnutrition +APO and dAUC% CT *vs.* Malnutrition by Student's *t*-test.

Hypertension might be associated with increased oxidative stress. To investigate the role of superoxide anion generated by NADPH oxidase activity on vascular reactivity to phenylephrine in post-weaning protein malnutrition rats, the NADPH oxidase inhibitor apocynin (0.3 mM) was used. Incubation with apocynin reduced reactivity to phenylephrine in all groups (Figure 4A and 4B), but the effect was greater in the post-weaning protein malnutrition group, as shown by the differences of the area under the concentration–response curves (Figure 4C). These results suggested that the post-weaning protein malnutrition increased the release of superoxide anion generated by NADPH oxidase.

Indomethacin (10 µM), a cyclooxygenase inhibitor, was used to investigate the putative role of prostanoids on vascular reactivity to phenylephrine in post-weaning protein malnutrition rats. Indomethacin did not alter the phenylephrine responses in aortic segments from both groups (results not shown).

Because vascular reactivity in the conductance arteries was almost unaffected, we also investigated the effect of post-weaning malnutrition on the vascular reactivity of the resistance arteries (tail artery). This protocol was used to obtain information regarding the effects of post-weaning protein malnutrition on the resistance vasculature.

**Figure 5:**
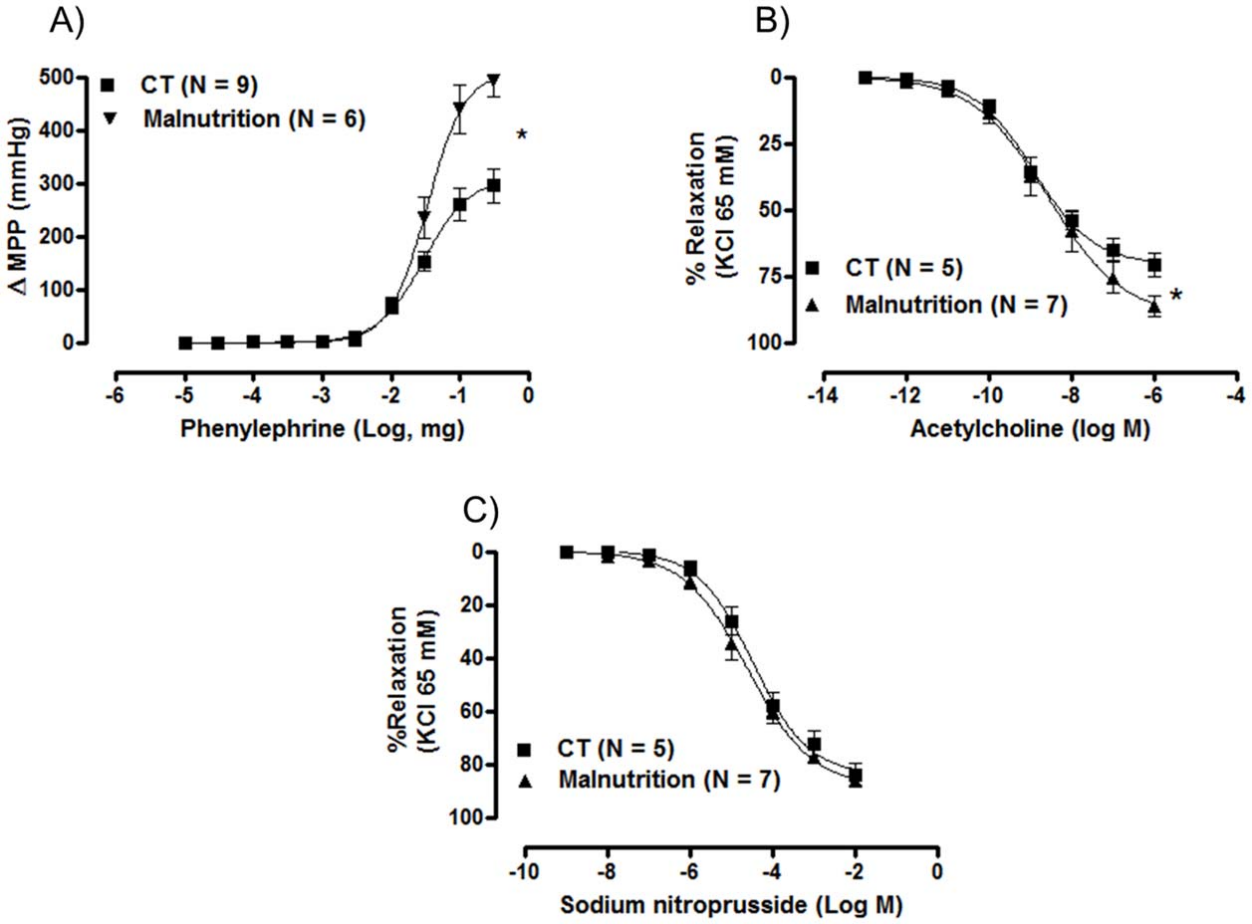
(A) Changes in the mean perfusion pressure (MPP) produced by phenylephrine (PHE) in tail vascular beds from the control (CT) and post-weaning protein malnutrition groups (Malnutrition). (B) Concentration-response curves produced by acetylcholine (ACh) in the tail vascular beds previously contracted with potassium chloride (KCl, 65 mM) (C) Concentration-response curves produced by sodium nitroprusside (SNP) in the tail artery bed previously contracted with with potassium chloride (KCl, 65 mM). The number of preparations is indicated in parenthesis. The results are expressed as the means ± SEM. * *P*<0.05 for Rmax: CT *vs.* Malnutrition, Student's *t-*test.

In the endothelium-intact tail vascular bed preparations from the post-weaning protein malnutrition group, the mean perfusion pressure was similar in both groups, as observed from the initial dose of phenylephrine response curve, which are similar (Figure 5A). However, the maximal response to phenylephrine (Figure 5A) was greater (493±29.5 mmHg, P<0.05, n = 10) compared to the CT group (295±32 mmHg, P<0.05, n = 10). In tail arteries previously contracted with KCl 65 mM, the acetylcholine induced-relaxation (Figure 5B) was modestly greater in the post-weaning protein malnutrition group (86.5±3.9%, mmHg, P<0.05, n = 10) compared to the CT group (70.8±4.2%, mmHg, P<0.05, n = 10). The endothelium-independent relaxation induced by sodium nitroprusside was similar in both groups (Figure 5C).

## Discussion

This study demonstrated that post-weaning protein malnutrition increased blood pressure, which was accompanied by increased vascular reactivity in resistance arteries (tail artery). However, in conductance arteries (aortic rings), malnutrition did not change the vascular reactivity due to increased basal release of NO, probably resulting from eNOS overexpression, although there was an increased release of free radicals derived from NADPH oxidase. Therefore, this balance prevented apparent changes in the phenylephrine concentration-response curves.

In this study, we investigated the effects of post-weaning protein malnutrition on blood pressure and vascular reactivity of resistance and conductance arteries in rats to elucidate some mechanisms that are correlated with two relevant issues in public health: malnutrition and hypertension. Post-weaning protein malnutrition was induced by a diet that reproduces a regional basic diet (RBD) [9], [10]–[12] widely consumed by inhabitants living in an area of sugar cane plantations along the coast of the State of Pernambuco, Brazil. This diet has been associated both with lower weight gain and the malfunction of various organs, probably because of its low protein content and vitamin and mineral deficiencies [9], [10]–[12]. It is important to mention that this diet is deficient in a qualitative rather than a quantitative nature, the latter of which is used by most researchers and represents the real consumption of this population. Depending on the region and season, populations in northeast Brazil and in other regions of the world might consume a similar diet. Therefore, the importance of knowing its effects on the organism might help to introduce future corrective interventions to minimize the risk of diseases associated with malnutrition.

Animals submitted to post-weaning protein malnutrition showed a marked reduction in weight gain. These results highlight the importance of nutritional status in the growth phase of the animal. They show a greater impact of the post-weaning protein malnutrition period including the stage of growth and development into adulthood. Similar results were previously observed [15] with use of the same diet. This reduction was also found in other studies using a rat experimental model induced by a dietary restriction of 50% [9], [16]–[19] or a low-protein diet with 6% protein [20], [21].

An alteration in body weight is an indicator of the nutritional status of the individual and might be associated with alterations in the cardiovascular system [22], [23]. Indeed, in our study we observed that the malnutrition triggers weight defect and hypertension. Several authors that have studied malnutrition induced during pregnancy in rats using quantitative models [18], [24]–[26] have shown that a decrease in body weight could be related to hypertension in the offspring. A previous report [27] has shown that the intrauterine malnutrition with a low protein (8% casein) diet in rats promotes an increase in blood pressure of the offspring at a young adulthood stage. Previous reports evaluating the effects of protein-caloric deprivation in rats have suggested an increased hypothalamic-pituitary-adrenal response and circulating catecholamines [28]–[30]. Then, the increase in heart rate and blood pressure might be related to increased sympathetic nervous system activity [31].

In accordance to these studies, Sawaya et al. [3] have demonstrated that in malnutrition, children might have increased diastolic blood pressure. Therefore, because the diastolic blood pressure reflects, in part, the peripheral vascular resistance, vascular reactivity might be altered in malnutrition conditions. Indeed, in our experiments, a qualitative model of malnutrition increased systolic and diastolic blood pressure and heart rate. For this purpose, we investigated the vascular reactivity of conductance (aorta artery) and resistance arteries (tail artery).

Malnutrition did not change the vascular reactivity in the isolated aortic rings. However, in the presence of L-NAME, a non-specific inhibitor of NOS, there was a greater vasoconstrictor response to phenylephrine in post-weaning protein malnutrition group, suggesting increased NO production. However, the endothelium-dependent relaxation induced by acetylcholine was similar in aortic rings isolated from controls and post-weaning protein malnutrition, suggesting that the oxide nitric release induced by acetylcholine was not modified.

As mentioned above, we observed that the malnutrition triggers hypertension. Hypertension might be associated with reduced nitric oxide bioavailability, although there are studies that have shown an increase in this vasodilator as a compensatory mechanism [32]–[34]. Moreover, other studies have shown that the synthesis and/or release of nitric oxide in arterial hypertension is normal, but its bioavailability is reduced due to increased superoxide anion production, mainly by increasing NADPH oxidase activity, which inactivates nitric oxide generating nitrite peroxide [35]–[37]. We then investigated the role of free radicals by blocking NADPH oxidase with apocynin. Our results suggested an increased release of superoxide anion derived from NADPH oxidase activity in post-weaning protein malnutrition arteries.

Franco et al. [17] also have shown that intrauterine malnutrition increases oxidative stress, suggesting a potential explanation for endothelial dysfunction development. Also, in the same study, they concluded that intrauterine malnutrition induces hypertension in both male and female offspring and hypertension may be more severe in males than females. Franco et al. [17] concluded that malnutrition alters endothelium-dependent responses and endothelial dysfunction is associated with decreased eNOS activity and expression in the aortas of the offspring. However, in our study, we showed increased nitric oxide release, which could result from an increase in eNOS protein expression. It's possible that the low-protein diet used in our study has lower arginine levels. Therefore, the increase of the eNOS protein levels, observed in the aorta, could be an adaptive response to the low arginine, and, consequently, low NO levels [37]. However, although the eNOS activity was not measurement, we observed an increase nitric oxide release in response to phenylephrine in isolated aortic rings. Therefore, in this study we cannot speculate that the increase in eNOS protein levels has relationship with the levels of arginine. In accordance with our findings, we might suggest that the increase of eNOS protein levels and nitric oxide release in response to phenylephrine in isolated aortic rings might be a compensatory mechanism induced by increased blood pressure or oxidative stress.

These results might be a compensatory mechanism induced by increased blood pressure or oxidative stress. The increase in superoxide anion, the most important free radical generated by NADPH oxidase activity, could decrease nitric oxide bioavailability [38]. The main sources of superoxide anion that are implicated in the genesis of endothelial dysfunction are xanthine oxidase and NADPH oxidase [38]. Particularly, the increased NADPH oxidase activity has been reported as a major source of superoxide anion in the vessel wall in experimental hypertension models [38].

Therefore, in order to investigate if the post-weaning malnutrition per se or the increment of blood pressure that was observed after the treatment with RBD could increase the release of free radicals, apocynin was incubated in isolated aortic rings from both groups. Based on the effects of apocynin on reducing phenylephrine reactivity, our findings point towards this possibility. Therefore, we believe that post-weaning protein malnutrition increases oxidative stress that is initially compensated by increased nitric oxide production. However, for a long period, this compensatory response can be lost, and the endothelial dysfunction might increase or maintain hypertension.

Because the vascular reactivity to phenylephrine in conductance arteries was not modified after the post-weaning malnutrition, other endothelium-derived vasoconstrictors are probably released. Therefore, we investigated the prostanoids derived from the cyclooxygenase pathway. In the presence of indomethacin, a COX inhibitor, there was no difference in the vasoconstrictor response to phenylephrine in the post-weaning protein malnutrition and control groups, suggesting that vasoactive prostanoids did not seem to be altered in this artery after malnutrition.

Considering that findings obtained from the aortic rings did not provide a clear explanation for the increased blood pressure, we investigated the effects of malnutrition on a resistance vessel. In fact, we demonstrated that in the tail artery of the post-weaning protein malnutrition group, the vascular reactivity to phenylephrine increased. Therefore, this increase in vascular reactivity might increase vascular resistance, which increases diastolic blood pressure. However, in the same artery, we observed that the relaxation response to acetylcholine increased. This effect could be a compensatory mechanism to the increased vascular reactivity or blood pressure.

Therefore, our results used a qualitative malnutrition model with low-protein to show an increase in blood pressure in the post-weaning protein malnutrition group, which was accompanied *in vitro* by an increase in vascular reactivity in the resistance arteries. However, in the conductance arteries, malnutrition did not modify the vascular reactivity probably due to the increased release of nitric oxide, which balances free radicals and prevents changes in the phenylephrine concentration-response curves. We suggest that the increase in free radicals and nitric oxide in the aortic rings of post-weaning protein malnutrition rats might be a compensatory mechanism to the increase in blood pressure.
